# CPNE3 moderates the association between anxiety and working memory

**DOI:** 10.1038/s41598-021-86263-6

**Published:** 2021-03-25

**Authors:** Chunhui Chen, Ziyi Wang, Chuansheng Chen, Gui Xue, Shuzhen Lu, Hejun Liu, Qi Dong, Mingxia Zhang

**Affiliations:** 1 State Key Laboratory of Cognitive Neuroscience and Learning & IDG/McGovern Institute for Brain Research, Beijing Normal University, Beijing, China; 2 Department of Psychological Science, University of California, Irvine, CA USA; 3 CAS Key Laboratory of Behavioral Science, Institute of Psychology, Beijing, China; 4 Beijing Key Laboratory of Brain Imaging and Connectomics, Beijing Normal University, Beijing, China; 5 Center for Studies of Psychological Application, School of Psychology, South China Normal University, Guangzhou, China

**Keywords:** Genome-wide association studies, Human behaviour

## Abstract

Mutual influences between anxiety and working memory (WM) have been extensively studied, and their curvilinear relationship resembles the classic Yerkes-Dodson law of arousal and performance. Given the genetic bases of both anxiety and WM, it is likely that the individual differences in the Yerkes-Dodson law of anxiety and WM may have genetic correlates. The current genome wide association study (GWAS) enrolled 1115 healthy subjects to search for genes that are potential moderators of the association between anxiety and WM. Results showed that *CPNE3* rs10102229 had the strongest effect, *p* = 3.38E−6 at SNP level and *p* = 2.68E−06 at gene level. Anxiety and WM had a significant negative correlation (i.e., more anxious individuals performed worse on the WM tasks) for the TT genotype of rs10102229 (resulting in lower expression of CPNE3), whereas the correlation was positive (i.e., more anxious individuals performed better on the WM tasks) for the CC carriers. The same pattern of results was found at the gene level using gene score analysis. These effects were replicated in an independent sample (N = 330). The current study is the first to report a gene that moderates the relation between anxiety and WM and potentially provides a genetic explanation for the classic Yerkes-Dodson law.

## Introduction

Working memory (WM) refers to the limited capacity system for temporary maintenance and manipulation of information^1,2^. Previous research has found that the level of anxiety is a major factor influencing WM^3–5^. A close link between WM and anxiety is also consistent with the finding that WM deficit and anxiety are co-symptoms of psychiatric disorders such as schizophrenia and attention deficit hyperactivity disorder. Researchers have speculated about two possible mechanisms linking anxiety to WM. Some researchers proposed that anxiety may decrease attentional control and the executive process, and thus impair the ability to maintain relevant information and inhibit irrelevant information^4,6^. Others suggested that because both anxiety and WM rely on prefrontal and parietal regions in the brain, they compete for the limited neural resource^7^.

Not all studies, however, have been consistent. Some studies found no significant correlation between anxiety and WM^8–10^. Some other studies even found that anxiety improved WM^11,12^. One possible explanation of these inconsistent results is the type of anxiety examined. For example, in a meta-analysis of 177 samples (22,061 individuals), Moran^13^ found that self-reported measures of anxiety were reliably related to poorer WM performance, but experimentally induced anxiety (e.g., watching emotional video clips) were not consistently associated with WM performance. Another possible moderator of the relation between anxiety and WM is the level of anxiety. When the anxiety level is in the low-to-moderate range, the association may be positive, but when the anxiety level is in the moderate-to-high range, the association may be negative, following the classic Yerkes-Dobson law for arousal and performance^14,15^.

Individual differences in WM and anxiety have their genetic bases. More than 100 genes have been reported to be associated with WM according to PhenoPedia (https://phgkb.cdc.gov/PHGKB/startPagePhenoPedia.action), many of which are genes related to neurotransmitters (e.g. dopamine, serotonin). However, most of the studies included in that database were candidate genes studies with small sample sizes and a loose statistical threshold (p < 0.05). A review of genome wide association studies (GWAS) on WM concluded that, although each study emphasized different genes, all of them are neuronal excitability-related genes, either related to ion-gated channels or to prefrontal dopamine activity^16^. Similarly, many genes were found to contribute to anxiety. A recent review summarized that neurotransmitter-related genes (e.g. *SLC6A4, COMT, MAOA, RGS2*) are also the most often studied candidate genes for anxiety-related phenotype. However, these candidate genes studies have been criticized for their small sample sizes and poor replicability, with the field moving towards large sample GWAS. GWAS on anxiety, however, reported different significant loci and did not reach a consistent conclusion^17^.

The curvilinear relationship between WM and anxiety may also be modulated by genes, but no study has tested this possibility. The current study searched the whole genome for genes that may interact with anxiety to affect WM.

## Method

### Subjects

We recruited 1135 healthy students (611 female, 21.2 ± 2.2 years old) from Beijing Normal University as the discovery sample. Twenty subjects were excluded due to the lack of data on anxiety or WM, so the final sample size was 1115 (602 female, 21.1 ± 2.2 years old). We also used a previously collected smaller dataset as the replication sample. The replication sample had 330 college students (191 females, 20.4 ± 0.9 years old) who had complete genotype and behavior data similar to the current study.

All subjects were healthy Han Chinese and self-reported not having a neurological or psychiatric history. This study was approved by the Institutional Review Board (IRB) of the State Key Laboratory of Cognitive Neuroscience and Learning at Beijing Normal University, and was performed in accordance with relevant guidelines and regulations. All subjects signed written informed consent forms and were paid for participation.

### WM measurement

A 3-back task was used to measure WM in the discovery sample^18^. N-back task was widely used in literature and showed good psychometric properties^19,20^. A series of 13 letters were presented on the screen one by one, shown for 750 ms followed by a blank screen of 2250 ms. Letters were randomly selected from the set (a, c, e, g, i, k, p, t, v). Subjects had to judge if the current letter was the same as the one presented three items earlier, and to make a response within 3 s. Both accuracy and speed were emphasized. Subjects practiced three series or until they reached an accuracy of 70%, to make sure they understood the task but were not over-trained. The formal experiment consisted of six series. The overall accuracy of six sequences were used to index WM performance, with higher accuracy representing better WM.

The replication dataset used three 2-back tasks to measure WM^21^. The most similar to that of the discovery sample was the task that used a series of 12 Tibetan characters. They were presented one by one, shown for 750 ms followed by a blank screen of 2250 ms. Subjects had to judge if the current character was the same as the one presented two items before, and to make a response within three seconds. Both accuracy and speed were emphasized. Subjects also practiced in the same way as the discovery sample. Subjects finished four series of the task and the overall accuracy was used to index WM performance.

### Anxiety measurement

Both samples used the Beck Anxiety Inventory (BAI) to measure anxiety^22^. Twenty-one anxiety symptoms were listed, and subjects were asked to rate to what extent they showed the symptoms over the past week on a four-point scale ranging from 0 (“not at all”) to 3 (“severely—it bothered me a lot”). Scores of all items were summed, with higher scores representing higher levels of anxiety. The Cronbach's α of BAI in this study was 0.920 in the discovery sample and 0.89 in the replication sample.

### Genotyping

This dataset was reported with previously^18^. 1–2 μg genome DNA (gDNA) was extracted from 250 μl blood using Axypre Blood Genomic DNA Kit (Corning Life Sciences cat.no.11313KC3). The concentration of all gDNA was quantified with the Qubit2.0 Fluorometer (Life Technologies, cat.no.Q32866) and the Qubit dsDNA HS Assay Kit (Life Technologies, cat.no.Q32854). For the discovery sample, 771 samples were genotyped on the Infinium Human Omni-Zhonghua-8 chips, 8 samples were genotyped on the Infinium Human Omni2.5-8 exome chips, and 336 were genotyped on Infinium OminiExpress-12 chips (Illumina, San Diego, CA, USA), all according to the manufacturer’s specifications. Genotyping module of Genome studio v3.0 (Illumina, San Diego, CA, USA) was used to call the genotypes based on the fluorescent signal with standard cluster algorithm. The replication samples were genotyped with Affy 6.0 chips according to the manufacturer’s protocol and genotypes were called using Genotyping Console 4.1.4. Samples with call rate less than 98% (four of the discovery sample, with genotype call threshold of 0.15) were re-genotyped thus all passed this threshold in the final dataset. Further data cleaning was performed separately for each kind of chips, using the following criteria in PLINK2 (https://www.cog-genomics.org/plink/1.9/)^23,24^: SNPs with missing data on more than 5% samples, or HWE *p* < 1E−6, or MAF < 0.01 were excluded, and subjects missing more than 5% SNPs were discarded too (although no subjects were excluded by the latter threshold).

Autosome genotypes of all chips were then imputed separately using Michigan Imputation Server (https://imputationserer.sph.umich.edu/index.html) following their protocol: (1) HRC tools (http://www.well.ox.ac.uk/~wrayner/tools) were used to check strand and to flip to forward strand when necessary; (2) data were transformed to VCF files and sorted for each chromosome; (3) data were uploaded to the server, and imputed using 1000G Phase 3 EAS population as reference. Imputed data were cleaned using home-made codes, and only SNPs with the imputation quality of *r*^2^ > 0.8 and MAF > 0.05 were retained. Then the three illumina datasets for discovery were merged and cleaned again (MAF > 0.05, HWE > 1E−6), retaining 4,856,474 SNPs. The replication Affy samples retained 4,706,674 SNPs. No duplicated or related subjects were identified (maximum PI_HAT = 0.0537, calculated with PLINK2).

### Statistical analysis

Descriptive analysis and correlation analysis of WM and anxiety were performed using SPSS 22.0. Genome-wide interaction was performed using PLINK2 linear regression, with WM as phenotype, and anxiety, gene, and anxiety by gene interaction as regressors. There was no obvious population stratification with this sample^18^, so the first two principal components of the genome, as well as gender and age, were included as the covariates. Additional analyses were conducted to include additional components (up to 10 total components), but the results remained the same (data not shown). Results are shown with LocusZoom (http://locuszoom.org/)^25^. Main and interaction p values from Plink2 were inputted to MAGMA^26^ for gene-level analysis. Gene definition was downloaded from the MAGMA website (https://ctg.cncr.nl/software/magma), using the NCBI37.3 version, resulting in 17,287 genes. The sum of –log(*p*) within a gene was calculated as the gene-level statistics (MAGMA default model). Significance threshold at the gene level was set to Bonferroni corrected p < 0.05/17,287 = 2.89E−06.

A gene score was calculated to represent the gene effect of significant gene(s). For a given significant gene, SNPs within the gene region were first clumped with PLINK2 (-clump-p1 5E-2 -clump-p2 5E-2 -clump-r2 0.50 -clump-kb 250) to remove bias induced by high LD and kept the most significant ones. The gene score was calculated by multiplying genotype (coded as 0/1/2) of these significant SNPs with their corresponding effect sizes (betas of the interaction terms in PLINK results) and then summing them up. A post hoc power analysis was conducted on the gene score effect using Gpower 3.1.9.7^27^. We used the F test to evaluate the power of detecting increased R^2^ by the interaction term in a linear multiple regression, as modeled in PLINK2.

To further explore possible biological mechanisms, we searched two databases for gene expression information: BrainSeq Consortium (http://eqtl.brainseq.org/phase1/eqtl/) and the Genotype-Tissue Expression (GTEx) database (http://gtexportal.org/). BrainSeq provides information about the association between genotype and RNA sequencing with data collected with postmortem DLPFC tissue of 175 schizophrenia patients and 237 controls. We report the eQTL results with the whole sample as recommended by the developer of the database (personal communications, October 2, 2019), although a separate analysis with data from the controls only showed the same results. The GTEx has expression data on issues from about 200 whole or partial brains, was supported by the Common Fund of the Office of the Director of the National Institutes of Health, and by NCI, NHGRI, NHLBI, NIDA, NIMH, and NINDS. The data used for the analyses described in this manuscript were obtained from the GTEx Portal on 09/15/20.

## Results

Mean accuracy of the WM task was 0.80 ± 0.11 for the discovery sample and 0.88 ± 0.07 for the replication sample, suggesting that subjects performed well on their respective WM tasks. The average score of anxiety was 5.11 ± 6.85 for the discovery sample and 11.32 ± 8.3 for the replication sample. These scores were low because the samples were healthy young students, with only a few subjects reporting moderate or severe symptoms (data points shown in Fig. 2). The bivariate correlation between anxiety and WM was not significant for either the discovery sample (*r* = − 0.05, *p* = 0.11) or the replication sample (*r* = − 0.07, *p* = 0.22).

**Figure 1:**
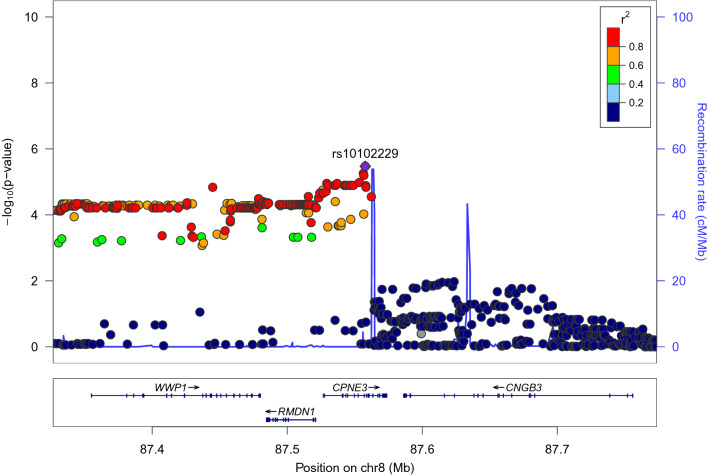
Interaction effects of SNPs with anxiety on WM within + /− 200 kb of rs10102229, plotted by LocusZoom. Y axis of each dot represents the interaction p value and the dots’ color represents LD with rs10102229.

In the discovery sample, the most significant SNP that interacted with anxiety to affect WM was rs10102229, with a *p* value of 3.38E−6. This SNP is located on an intronic region of *CPNE3* gene (see supplementary Figure S1). At the gene level, MAGMA showed a significant effect of *CPNE3*, *p* = 2.68E−06, which survived Bonferroni correction (0.05/17,287 = 2.89E−06). As shown in Fig. 1, many SNPs within this region had effects with *p* < 1E−4. This is consistent with the quantitative trait loci (QTL) idea that many loci had relatively small effect sizes but their cumulative effect is strong, which can be captured by gene-level analysis. No SNPs or genes showed significant main effect on WM after multiple comparison correction, but the main effect of *CPNE3* had a significance level of p = 1.03E−03. MAGMA results at the gene level can be found in supplementary Table S1.

Three SNPs (rs10102229, rs1866905, rs11782610 -) were identified as tag SNPs to calculate a gene score for the *CPNE3* gene. This gene score and anxiety had a highly significant interaction effect on WM (*p* = 2.14E−7). The post hoc power analysis showed that given the effect size (the ratio of the proportion of variance explained by the interaction term over the error variance) was 0.025, the power of finding this effect with a sample size of 1115 at *α* < 2.89E−06 (with a stringent Bonferroni correction) was 0.71. This interaction effect was replicated in the replication sample by MAGMA gene analysis (*p* = 0.039 for *CPNE3*) as well as the gene-score-by-anxiety interaction analysis (*p* = 0.0016).

**Figure 2:**
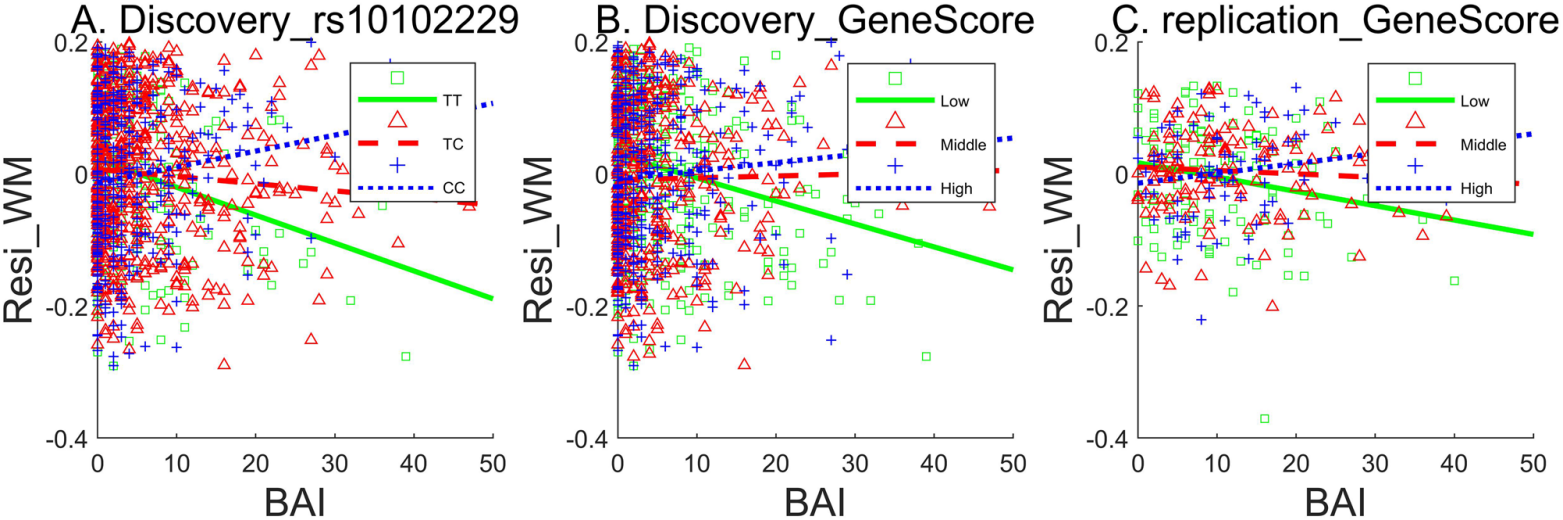
Interaction of rs10102229/ gene score and anxiety on WM. The X axis represents anxiety and the Y axis represents WM residual after controlling first two principal components of the genome, gender and age.

To investigate the nature of this interaction effect, we calculated correlations between WM and anxiety separately for subjects with CC, CT, and TT genotypes on rs10102229. There were 329 CC, 561 TC, and 225 TT individuals in the discovery sample, whose distribution was in Hardy–Weinberg equilibrium (*p* = 0.88). As shown in Fig. 2A, the TT group showed a negative correlation (*r* = − 0.26, *p* = 8.26E−5), with higher anxiety being linked to impaired WM; the CC group showed a positive correlation (*r* = 0.14, *p* = 0.01), with higher anxiety being linked to better WM performance; and the TC group showed no significant correlation between WM and anxiety (*r* = − 0.07, *p* = 0.10).

To explore the interaction effect at the gene level, we divided subjects into low, medium, high gene score groups with approximately the same group size (n = 373/383/359 respectively, not exactly the same size, because many individuals had the same gene scores). In the discovery sample, the low gene score group showed a negative correlation between anxiety and WM (*r* = − 0.24, *p* = 4.48E−6), the other two groups showed no significant correlation (*r* = 0.02, *p* = 0.77, *r* = 0.08, *p* = 0.14 for medium and high gene score groups) (Fig. 2B). The same trend was found in the replication sample (*r* = − 0.21/− 0.07/ 0.20, *p* = 0.02/0.43/0.08, n = 121/128/81, for the low/medium/high gene score groups, respectively, Fig. 2C).

BrainSeq shows that rs10102229 is strongly associated with transcripts of CPNE3 (minimum *p* = 6.1E−21), RMDN1 (minimum *p* = 1.3E−95), WWP1 (minimum *p* = 4.8E−21) (supplementary Table S2), with TT homozygotes showing the lowest level of expression of these genes (see BrainSeq website). Similarly, the GTEx shows that TT homozygotes are associated with lowest expression level in brain tissues, minimum *p* = 4.6E−3 in the nucleus accumbens for CPNE3, minimum *p* = 4.3E−23 in the cerebellum for RMDN1, and minimum *p* = 0.05 in the spinal cord forWWP1. As we can see from Fig. 1, there is a long LD block at the upstream of rs10102229 covering these three genes. The interaction effects for *RMDN1* and *WWP1* at MAGMA gene level had *p* = 4.3E−5 and 4.6E−5 respectively in the discovery sample, both of which were relatively strong but did not survive Bonferroni correction, and both were replicated in the replication sample (*p* = 0.011 and 0.019 respectively). Therefore, it seems possible that *RMDN1* and *WWP1* played a role in the interaction effect we found in this study.

## Discussion

The current study found that *CPNE3* interacted with anxiety to affect working memory, and replicated the finding in an independent sample. Rs10102229 had the strongest effect, and nearby genes *RMDN1* and *WWP1* in strong LD also showed moderate effects. As we noted in the introduction section, the relationship between WM and anxiety has been widely studied but the results are inconsistent. One possible reason is their curvilinear relationship akin to the Yerkes-Dodson law regarding arousal and performance. Here we suggested that this relationship may have a genetic basis, with some individuals of a particular genetic type (the CC genotype) showing a positive relationship and others showing a negative correlation (the TT genotype) or no correlation (the heterozygotes).It should be mentioned that the Yerkes-Dodson law was based on experimental evidence, where the arousal level was experimentally manipulated and was relative between conditions (either within- or between-subjects design). There is no direct correspondence between “high” arousal in those experiments and “high” anxiety based on a trait anxiety measure such as BAI. Given the ethical concerns in experiments, it is likely that “high” arousal in experiments may not, and should not, reach the clinical level of anxiety disorder. Our study was not designed to calibrate the level of state anxiety in experiments and the level of anxiety as a trait, but rather to entertain the possibility that genetic factors may lead to opposite associations between anxiety and working memory. Future research is needed to experimentally test whether subjects with a given genotype (i.e., CC genotype on rs10102229 or high gene score on *CPNE3*) would show a higher threshold of arousal for it to negatively impact their performance on various tasks.

We would like to point out that neither the correlation between anxiety and WM nor the main effect of CPNE3 on WM was significant, suggesting the true action was the interaction between anxiety and CPNE3, which had not been tested before. There exist thousands of GWAS as assembled in GWAS Catalog (https://www.ebi.ac.uk/gwas/), but only a few genome-wide environment interaction studies. It is imperative to conduct the latter type of studies in order to reveal important mechanisms. These results suggested that for clinical practice or WM training studies, it may be of value to take into account the genotype of the participants.

*CPNE3* is a member of calcium-dependent membrane-binding protein family, and has often been reported to be involved in diseases such as acute myeloid leukemia, breast cancer, lung cancer, etc. Although this gene’s role in WM and anxiety has not been reported with direct evidence, Cohen, et al.^28^ found that *CPNE3* expressed transcripts with relatively shorter 3′UTRs in schizophrenia patients than in healthy controls. Numerous studies have found that schizophrenia patients show deficits in WM^29–33^ and suffered from anxiety^34–38^. According to BrainSeq and GTEx, the TT genotype of rs10102229 was associated with a lower expression of *CPNE3*, and we found that WM and anxiety showed a negative correlation in this group (i.e., higher anxiety was linked with worse WM), which was similar to the pattern of schizophrenia patients. Gene score analysis revealed the same pattern. Previous experimental research has consistently shown that a certain level of anxiety can promote motivation and enhance WM^4,6^. Our results showed that such promotive effects may be limited to individuals with certain genotypes (i.e., CC carrier of CPNE3). *RMDN1* and *WWP1* are in a high LD block with *CPNE3*. However, the effects of these genes on cognition and emotion are in need of direct or indirect evidence.

There are several limitations in the current study. First of all, this study enrolled healthy Chinese college students, whose results may not generalize to other populations or to samples with severe disorders. Second, our sample size was moderate compared to many whole-genome association studies, but we replicated the interaction effect in an independent sample. Third, the current study only used self-reported BAI questionnaire to measure anxiety symptoms during the past week, but not state anxiety. Considering the distinction between state and trait anxiety, future research should directly investigate whether CPNE3 consistently moderates the effects of self-reported trait and state anxiety (e.g., using the State Trait Anxiety Inventory) and experimentally induced anxiety on WM.

In summary, the current study searched the whole genome and found that *CPNE3* interacted with anxiety to affect WM, with rs10102229 showing the strongest effect. The TT genotype of rs10102229 results in lower expression of *CPNE3*, and participants with this genotype showed a negative correlation between WM and anxiety, and participants with the CC genotype showed a positive correlation, whereas those with the TC phenotype showed no significant correlation. The same pattern was found at the gene level as shown by gene score analysis. These effects were replicated in an independent sample. Further studies are needed to reveal the underlying biochemical mechanism.

## Supplementary information

Supplementary information.

Supplementary tables.

## Data Availability

All data are available on request.
