# Supplementary material for: CPNE3 moderates the association between anxiety and working memory
Source: Sci Rep. 2021 Mar 25;11:6891. doi: 10.1038/s41598-021-86263-6 (PMC7994849; doi:10.1038/s41598-021-86263-6)

**CPNE3 moderates the association between anxiety and working memory**

Chunhui Chen ^1,4*^, Ziyi Wang^1,5^, Chuansheng Chen^2^, Gui Xue^1^, Shuzhen Lu^1^, Hejun Liu^1^, Qi Dong^1^ , Mingxia Zhang^3*^

^1^State Key Laboratory of Cognitive Neuroscience and Learning & IDG/McGovern Institute for Brain Research, Beijing Normal University, Beijing, China

^2^Department of Psychological Science, University of California, Irvine, California, USA

^3^CAS Key Laboratory of Behavioral ScienceInstitute of Psychology BeijingChina

^4^Beijing Key Laboratory of Brain Imaging and Connectomics, Beijing Normal University, Beijing, China

^5^Center for Studies of Psychological Application, School of Psychology, South China Normal University, China

* Corresponding authors:

Chunhuichen: [chenchunhuichina@bnu.edu.cn](mailto:chenchunhuichina@bnu.edu.cn)

Mingxia Zhang: [zhangmx@psych.ac.cn](mailto:zhangmx@psych.ac.cn)

**Supplementary table S1.** Main effects of genes and interaction effects of genes by anxiety on WM calculated with MAGMA.

**Supplementary table S2.** Association between rs10102229 and gene expression searched and downloaded from BrainSeq Consortium.

**Supplementary Figure S1.** Manhattan Plot of whole genome by anxiety interactions on WM. SNPs are plotted on the x-axis based on their position on each chromosome and -log10 transformed p-value on the y-axis. Dotted line represents p=5E-6.


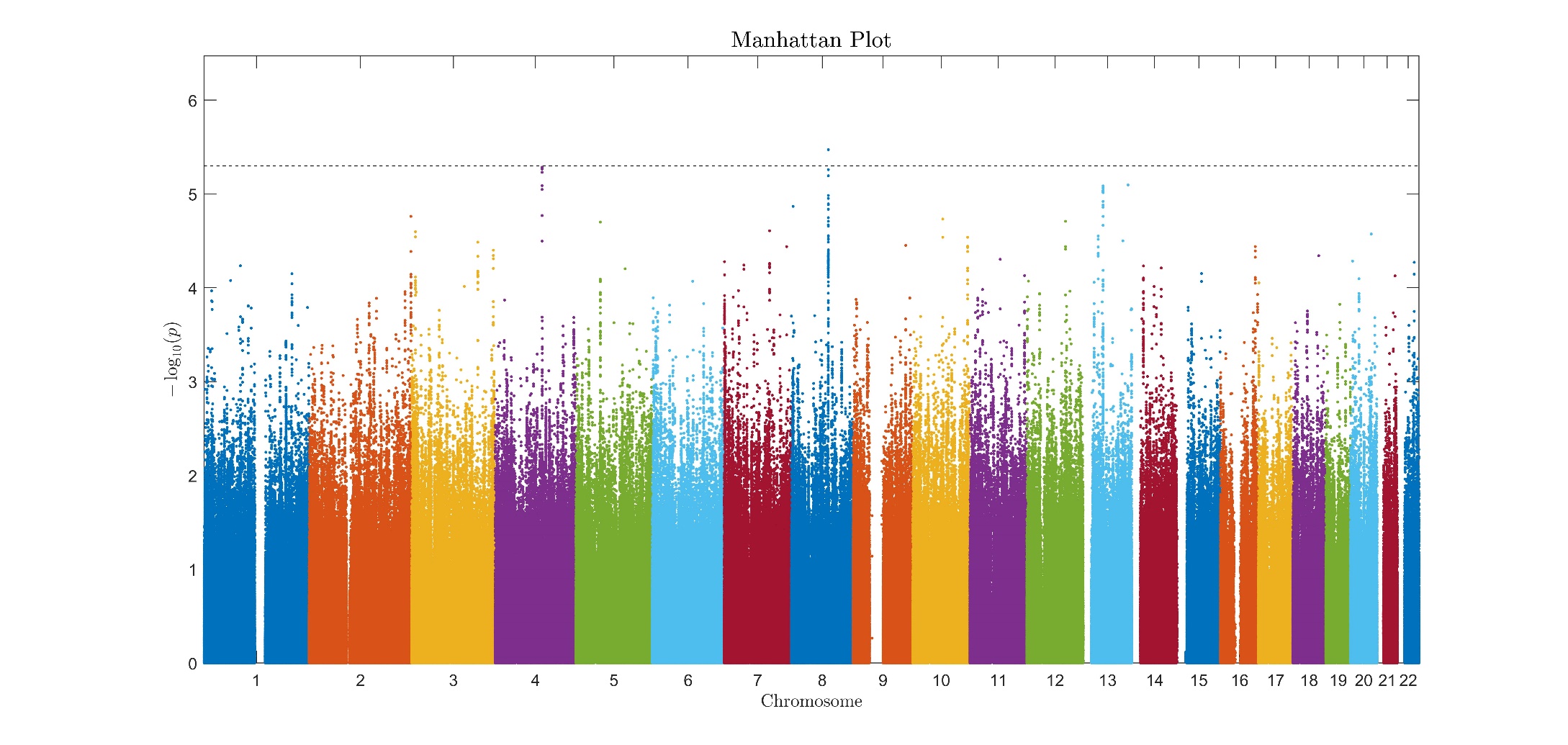

Supplement: Supplementary file 1 — Supplementary information. [file 41598_2021_86263_MOESM1_ESM.docx]
